# Predictions from deep learning propose substantial protein–carbohydrate interplay

**DOI:** 10.1073/pnas.2523342123

**Published:** 2026-05-18

**Authors:** Samuel W. Canner, Ronald L. Schnaar, Jeffrey J. Gray

**Affiliations:** ^a^Program in Molecular Biophysics, Johns Hopkins University, Baltimore, MD 21218; ^b^Department of Pharmacology and Molecular Sciences, Johns Hopkins University School of Medicine, Baltimore, MD 21287; ^c^Department of Neuroscience, Johns Hopkins University School of Medicine, Baltimore, MD 21287; ^d^Department of Chemical and Biomolecular Engineering, Johns Hopkins University, Baltimore, MD 21218

**Keywords:** proteome, protein–carbohydrate interactome, interactome, carbohydrate, glycan

## Abstract

Noncovalent interaction between proteins and carbohydrates (sugars, glycans) is the basis for biological functions from metabolic regulation to intercellular recognition. It is a grand challenge to identify the protein–carbohydrate interactomes in organisms. Direct experiments would require extensive libraries of glycans to distinguish binding from nonbinding proteins. Computational screening of proteins for carbohydrate binding potential provides an attractive alternative. Current estimates propose that <5% of proteins bind carbohydrates, a number that is not well established. We therefore developed a neural network, “Protein interaction of Carbohydrates Predictor” (PiCAP), to predict whether a protein noncovalently binds to a carbohydrate. We trained PiCAP on a manually curated dataset of known carbohydrate binders and proteins that we identified as likely *not* to bind carbohydrates (transcription factors, cytoskeletal components, and small-molecule-binding proteins). PiCAP achieves 90% balanced accuracy on protein-level predictions of carbohydrate binding/nonbinding. Using the same datasets, we developed Carbohydrate Protein Site Identifier 2 (CAPSIF2) to predict protein residues that interact noncovalently with carbohydrates. CAPSIF2 achieves a Dice coefficient of 0.57 on residue-level predictions on our independent test dataset, outperforming previous models. To demonstrate the models’ biological applicability, we investigated human cell surface proteins and further predicted the likelihood of carbohydrate binding in six proteomes (**Escherichia coli*, Mus musculus, Homo sapiens, Saccharomyces cerevisiae, Caenorhabditis elegans, Drosophila melanogaster*). PiCAP predicts that ~35 to 40% of proteins in these proteomes bind carbohydrates, with 75% of extracellular and cell surface proteins predicted to bind. The PiCAP predicted binders are enriched for functions including growth factor receptor binding, inflammation, and cell–cell adhesion.

## Significance

The totality of the protein–carbohydrate interactome remains elusive, in part due to the inability to directly probe a proteome versus a glycome in a high throughput manner. Here we show a high-throughput methodology to predict protein–carbohydrate interactions at proteomic scales by using structural and sequence information. To provide a better grasp of the role of carbohydrates in cellular functions, we created a computational method to predict the carbohydrate binding profiles of several proteomes (human, mouse, fly, nematode, yeast, and bacteria).

Carbohydrates (sugars, glycans) are a diverse class of molecules consisting of monosaccharides–individually and in glycosidic linkage as oligosaccharides and polysaccharides. In mammalian biology, carbohydrates are studied as two distinct families of molecules that are the focus of two disciplines. As metabolic precursors, from food or stored reserves, polysaccharides and monosaccharides (primarily glucose) are transported into and stored in the cytoplasm where they are subject to catabolic transformations to produce energy (1). In contrast, distinct covalent groupings of varied monosaccharide building blocks covalently bound to proteins (glycoproteins and proteoglycans) and lipids (glycolipids) are relatively stable and are abundant at the cell surface and in the extracellular milieu (2). A notable exception is O-GlcNAcylation, the reversible covalent attachment of the single sugar N-acetylglucosamine (GlcNAc) to serines and threonines of many cytoplasmic and nuclear proteins (3).

Like their structures, the functions of carbohydrates are diverse. Among other functions they play essential roles in metabolism, they contribute to protein, cell, and tissue structures, and they engage in molecular recognition upstream of cell–cell adhesion and cell regulation (2). Most of these functions involve engagement of glycans by proteins (4). Protein–carbohydrate interactions have predominantly been studied using chemical, biochemical, and structural biology methods, the latter of which reveal common features of carbohydrates that support protein recognition (2, 5). While there are many monosaccharide structures, the most abundant structures by far are pyranose rings (five carbons, one oxygen) surrounded by appended hydroxyl groups. Monosaccharide variety comes from the stereochemistry of the hydroxyls and the presence of additional select constituents around the ring, such as N-acetyl or carboxyl groups. Protein–carbohydrate recognition is most often driven by networks of hydrogen bonds surrounding CH-π stacking interactions between hydrophobic amino acid residues stacked precisely above and/or below the pyranose ring (5). This general molecular definition of protein–carbohydrate binding parameters is not, per se, directly applicable to high-throughput screening of genomes. This is where advances in the computational field may be directive, as explored in this paper (2). This mechanism-independent approach uses databases of known protein–carbohydrate interactions to build an algorithm to extend common carbohydrate binding features to the entire genome.

With the advent of the third generation of machine learning and large datasets, many novel algorithms have been created to better understand biophysical phenomena (6, 7). Deep learning methods have recently overtaken most traditional algorithms for all biomolecular methods on all biopolymers, including prediction of protein structure, protein-small molecule interactions, and de novo protein design (8–12). Two of the largest computational steps in biophysics made recently are the releases of AlphaFold 2 (AF2) (8) and ESM (13). AF2 revolutionized the protein structure landscape by creating a public, easily accessible, and accurate method for protein structure prediction. AF2 additionally predicted the protein structures of 48 organisms that are publicly accessible (10). ESM (named for evolutionary scale modeling) revolutionized protein sequence representations through its transformer architecture, with ability to richly encode the language of protein sequences (13).

Leveraging recent computational advances, scientists are beginning to explore the breadth of protein–carbohydrate interactions. We expect some of these protein–carbohydrate interactions to be involved in carbohydrate metabolism, some in intermolecular recognition and regulation of protein functions (e.g., O-GlcNAc), and others in cell adhesion and cell regulation. The goal of this work is to use computational advances to predict the protein–carbohydrate interactome: all proteins amenable to carbohydrate binding, in its broadest interpretation. Conventionally, researchers have focused on the carbohydrate binding protein family of lectins, which excludes enzymes, carriers, or native sugar sensors. Here we computationally explore carbohydrate-binding proteins without excluding them based on function; we expect to capture proteins across metabolic, structural, and molecular recognition functions. Although this approach is agnostic to carbohydrate species; as discovery progresses, our work may be expanded to provide further subcharacterization to identify the functional definitions of carbohydrate binding.

Recently we developed a dataset and two models, named CArbohydrate Protein Site IdentiFier (CAPSIF):Graph and CAPSIF:Voxel, to predict the protein residues involved in noncovalent carbohydrate–protein interactions (14). CAPSIF:V and CAPSIF:G are trained and tested on the same dataset and use the same residue level encodings, but CAPSIF:V encodes proteins onto a 3D voxelized grid with a UNet architecture whereas CAPSIF:G uses an equivariant graph neural network (EGNN) message passing framework; CAPSIF:V slightly outperformed CAPSIF:G by all measured metrics.

Since both CAPSIF models were released, two similar models have been created. Bibekar et al. released Protein Structure Transformer (PeSTo)-Carbs, which uses a geometric transformer architecture to predict residues involved in protein–carbohydrate interactions (15, 16). PeSTo-Carbs employs a query-key-value attention mechanism with message passing across atoms that are then pooled for residue-wise predictions (15). He et al. released DeepGlycanSite, which leverages a geometric message-passing architecture to predict a glycan binding site in both the case of a known ligand and an unknown ligand (16). PeSTo-Carbs modestly outperforms both CAPSIF models on all reported metrics, whereas DeepGlycanSite focuses on binding to nucleotide structures as compared to carbohydrate-only polymers.

Most carbohydrate–protein interaction algorithms rely on multiple datasets to extract experimental coordinates for prediction (14, 15). Currently the standard protein–carbohydrate dataset is UniLectin; however, UniLectin focuses only on proteins in the lectin family and thereby does not include other carbohydrate binding proteins (17). Recently, DIONYSUS was released detailing an immense set of experimental carbohydrate binding proteins with noncovalently bound carbohydrate (18).

Since experimentally solved structures can be difficult to obtain, especially in the presence of a carbohydrate ligand, some datasets of sequences exist that identify carbohydrate binding proteins. The Carbohydrate Active enZYmes (CAZY) dataset identifies sequences of catalytically active proteins that act on glycosidic bonds (19). LectomeXplore is a dataset that identifies known lectins, their associated structures (if known), and potential lectins as identified by sequence similarity via a hidden Markov model (HMM) (20). Rather than limiting their work to known lectins, Zhang et al. developed high throughput experiments with a ganglioside probe that identified 873 putative human proteins that likely interact with gangliosides (21). These works are limited by their scope, requiring either specific protein families or specific carbohydrate species to interact.

Here, we present frameworks to both predict whether a protein can bind to carbohydrates and where on that protein the carbohydrate binds, entitled Protein interaction of CArbohydrate Predictor (PiCAP) and CArbohydrate Protein Site IdentiFier 2 (CAPSIF2). Both models leverage a large dataset with two training stages, first using all small molecule binding interfaces and then fine-tuning with carbohydrate-specific data. We assess the ability of these models in their tasks. We then validate PiCAP against the work of Zhang et al. (21). and identify potential outliers in their dataset. Finally, we use these models to predict carbohydrate binding proteins, and the residues of these proteins for six model system proteomes. While these proteome-wide predictions are likely noisy, they define the broad scope of the problem and invite refinement by future experimental and computational methods.

## Results

### NoCAP: A Nonbinder Dataset.

Many datasets exist for protein–carbohydrate interactions, with the most notable being DIONYSUS (Table 1). However, there is no dataset of proteins that do *not* bind to carbohydrates; therefore, we developed a dataset consisting of proteins known to bind carbohydrates and proteins that likely do not bind carbohydrates based on biophysical intuition. Although the nonbinder dataset is likely mildly contaminated with some currently unknown carbohydrate binding proteins, we believe this dataset to be generally representative of proteins that do not bind carbohydrates. We denote this combined dataset as Nonbinder and binder of CArbohydrate Protein interactions (NoCAP) (Table 1). In addition, we created a subset of NoCAP, named DIONYSUS-Residue (DR) as all binding proteins in NoCAP with a bound ligand, retaining the DIONYSUS name as most protein structures were retrieved from the DIONYSUS dataset (Table 1).

**Table 1.** Experimental structural datasets

Dataset	NoCAP	DR	Description	n proteins
CAPSIF (14)	√	√	Bound protein–glycan complexes	802
TS 90	√	√	Test set for Pesto-Carbs; a subset of the CAPSIF test set	90
DIONYSUS (18)	√	√	Bound protein–glycan complexes	5,461
UniLectin (17)	√		Lectin structures and sequences	2,881
ProGen (23)	√		De novo designed lysozymes	69
Designed-NB (35)	√	√	List of crystal and complementary designed nonbinders	2,800
SAbDab (51)	√	√	Crystalized antibodies to their antigen (filtered)	2,925
PDB-Bind (53)	√	√	Small molecule binders (filtered to remove carbs)	17,191
PDIDB (62)	√		DNA-binding proteins (putative noncarb binders)	922
Manual selection	√		Biophysically putative nonbinding proteins (fatty acyl synthases, cytoskeletal components, flippases, ion transporters, ribosomal proteins)	606

Columns 2 and 3 indicate dataset inclusion in our NoCAP or DR datasets.

To provide a more comprehensive view of the physiologic characteristics of protein–carbohydrate interactions, we curated the datasets to contain complete structures (e.g., not separate chains) and incorporated both ligand-bound *holo* and unbound *apo* forms. While DIONYSUS already aggregated several sources including Unilectin3D and SabDAb, we additionally use Unilectin3D to obtain unbound *apo* structures of lectins and leverage SabDAb to access antibody-protein and antibody-nucleic acid complexes. In total, NoCAP contains 30,429 structures, with 9,509 carbohydrate binding proteins and 21,339 putative nonbinders. The DR set, which is that of bound protein–carbohydrate complexes, contains 6,263 structures in total.

### CAPSIF2 Outcompetes All Previous Models Identifying Carbohydrate-Binding Residues.

We constructed an equivariant graph neural network (EGNN) named Carbohydrate Protein Site IdentiFier 2 (CAPSIF2) leveraging the same general architecture of our previous work CAPSIF:Graph (CAPSIF:G). Although CAPSIF:G underperformed CAPSIF:V, we chose the EGNN architecture because it is scalable to proteins of any size, while CAPSIF:V is limited by the size of the underlying convolutional voxels. Although the dataset of this work (6,724 protein structures) is substantially larger than our previous work (~800 protein structures), there is still an intrinsic data imbalance in that most protein residues (~95%) do not bind carbohydrates. To address this, we once again leveraged the Dice loss (Table 2) to emphasize the residues that bind carbohydrates (*Materials and Methods*).

**Table 2.** Average metrics for each deep learning architecture on test sets

Model	DR Dice	DR MCC	TS 90 Dice	TS 90 MCC
CAPSIF2	**0.573**	**0.574**	0.616	0.607
Pesto-Carbs	0.493	0.492	**0.638**	**0.624**
CAPSIF:V	0.226	0.202	0.608	0.622

Dice coefficient is described as 2TP/(2TP + FP + FN), where TP, FP, and FN are the counts of the true positives, false positives, and false negatives, respectively. MCC is the Matthews correlation coefficient. Boldface indicates the best performance for each metric.

**Fig. 1.**
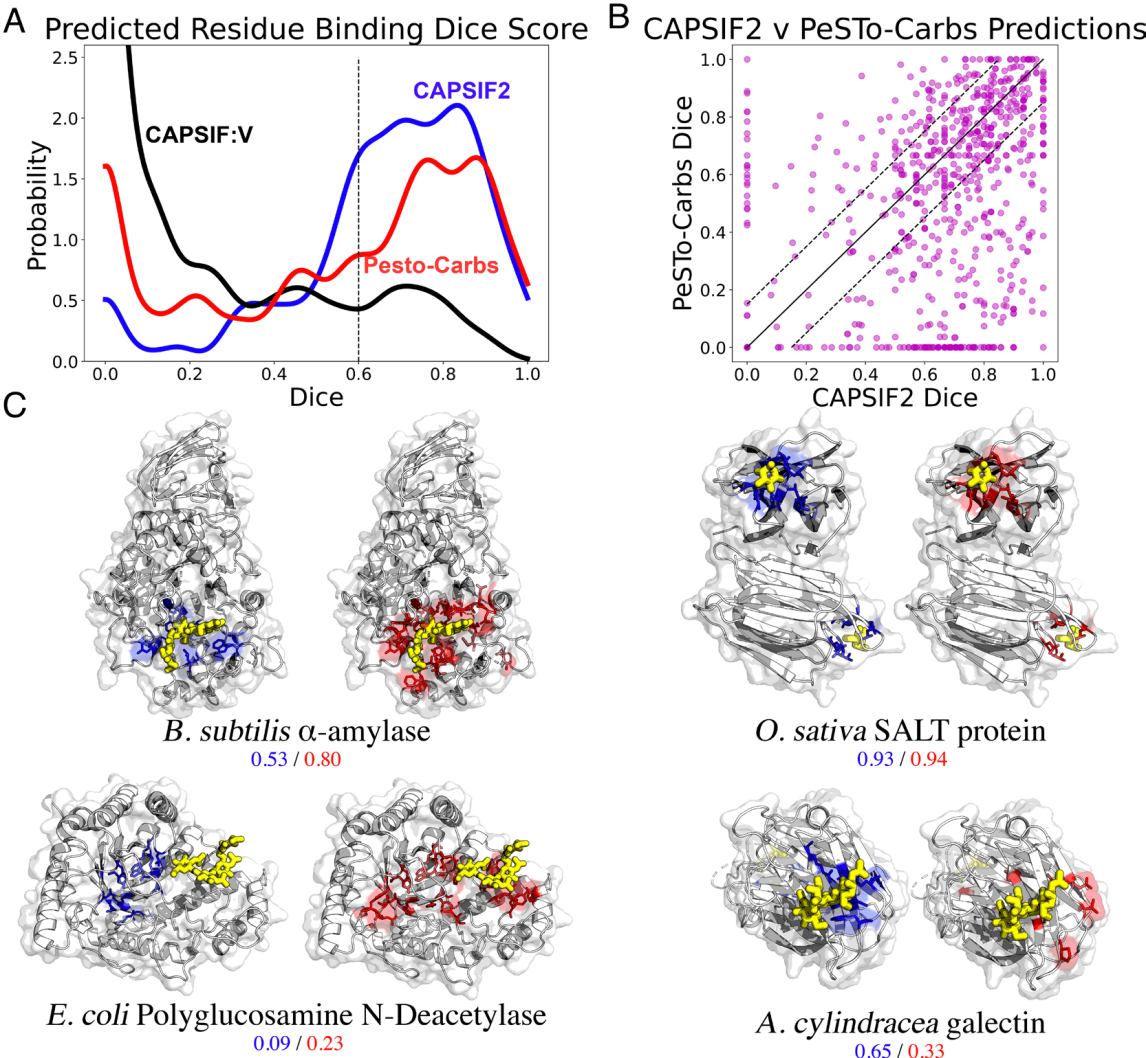
Comparison of CAPSIF2 and PeSTo-Carbs on residue-wise prediction tasks. (*A*) Distribution of Dice coefficient across prediction targets (proteins) for CAPSIF2 (blue), PeSTo-Carbs (red), and CAPSIF:V (black) on the DR test set. Densities smoothed with a Gaussian kernel density estimate (KDE, bandwidth *h =* 0.04). (*B*) Per-target comparison of CAPSIF2 to PeSTo-Carbs. (*C*) Side-by-side comparison of carbohydrate (yellow) bound proteins (gray) predictions by CAPSIF2 (blue, *Left*) and PeSTo-Carbs (orange, *Right*) on *B. Subtilis* α-amylase (1BAG), *O. sativa* SALT protein (5GVY), *Escherichia coli* poly-β-1,6-N-acetyl-D-glucosamine N-deacetylase C-terminal domain (4P7R), and *A. cylindracea* galectin (5XFD). Per-target Dice coefficients shown below.

In Fig. 1 and Table 2, we compare our results to PesTo-Carbs (15) and our previous model CAPSIF:V (14). On the TS-90 test set, CAPSIF2 achieves 0.616 Dice and 0.607 MCC metrics and PesTo-Carbs outcompetes our model on this test set with a 0.638 Dice coefficient and 0.624 MCC (Table 2). Contrarily on the DR test set, CAPSIF2 achieves 0.573 Dice coefficient and 0.574 MCC, while PesTo-Carbs only achieves 0.493 Dice and 0.492 MCC metrics. On a per target basis, CAPSIF2 performs greater than 0.15 Dice better than PesTo-Carbs on 40% of targets and PesTo-Carbs performs greater than 0.15 Dice than CAPSIF2 on 15% of targets (Fig. 1*B*).

We further show the results of specific targets in Fig. 1*C*. In most of these cases, PesTo-Carbs and CAPSIF2 can successfully find the binding region, with varying accuracy; however, they both appear to fail on some targets, such as N-acetyl-D-glucosamine N-deacetylase. This target notably has an observable pocket in the center of the structure, which CAPSIF2 and PesTo-Carbs incorrectly identify as the binding region, wherein the experimentally solved oligosaccharide is proximal to the pocket.

### PiCAP Accurately Predicts Carbohydrate Binding and Nonbinding on Experimental Structures.

Leveraging the same foundational network structure as CAPSIF2, we constructed the equivariant graph neural network (EGNN) named Protein interaction of Carbohydrate Predictor (PiCAP) with five additional layers to yield a single value prediction of whether a protein does or does not bind a carbohydrate. PiCAP assesses the spatial relationship of residues over an increasing context window, pooling the sequence into a fixed size 2D image, and providing a singular classification prediction based on that 2D representation. To our knowledge, PiCAP represents a previously unavailable DL approach to assess protein–noncovalent binding of carbohydrates at a protein level.

**Fig. 2.**
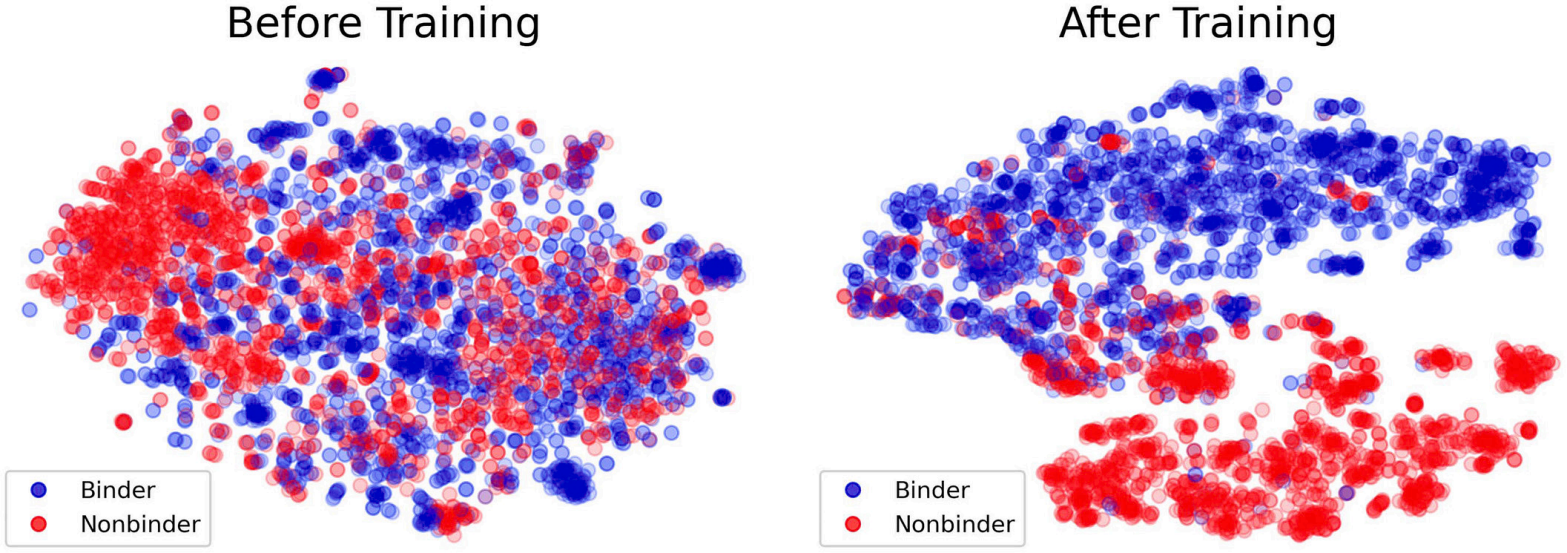
T-distributed stochastic neighbor embedding (T-SNE) diagrams of the PiCAP final layer embeddings of the NoCAP test set. (*Left*) The randomly initialized model’s final layer output. (*Right*) The final trained model’s final layer output.

We tested PiCAP on a holdout set based on sequence similarity, finding that PiCAP achieves an 89.6% balanced accuracy (BACC), with a 96.3% true positive rate (TPR) and 82.8% true negative rate (TNR) (Table 3). The ability to separate out carbohydrate binding (blue) and nonbinding (red) proteins is further demonstrated in 2D t-distributed stochastic neighbor embedding (T-SNE) plots (Fig. 2) (22). Despite our best efforts, we do expect that the nonbinder dataset is likely contaminated with some carbohydrate binding proteins, therefore we must further discriminate PiCAP’s ability to predict on specific test set subsets.

**Table 3.** Metrics for PiCAP on the NoCAP test set and associated subsets with the number of proteins in parentheses

Test Set	Accuracy
NoCAP BACC (4,380)	0.896
NoCAP TPR (2,343)	0.963
NoCAP TNR (2,037)	0.828
TNR Ribosome (7)	1.0
TNR Holdout (92)	0.902
TPR ProGen Lysozymes (69)	0.841
TNR Designed Nonbinders (186)	0.608
Antibody BACC (50)	0.562

BACC is balanced accuracy. TPR is True Positive Rate TPR = TP/(TP + FP). TNR is True Negative Rate TNR = TN/(TN + FN).

When inspecting subsets of NoCAP (Table 3), we find PiCAP correctly predicts all the protein chains of the ribosome assembly as nonbinders. We further have a holdout set of multiple proteins from various protein families, consisting of fatty acyl synthases, actin, myosin, and flippases, where PiCAP achieves an encouraging 90.2% accuracy on this negative subset. We observed that PiCAP performed well on designed lysozymes from the ProGen language model (23) with an 84.1% accuracy. This high accuracy may be a result from the high redundancy of the ProGen lysozymes, which span only five families. Contrarily, PiCAP achieves poor accuracy on computationally designed nonbinder proteins, these being poor designs regarded as nonbinding to the carbohydrate on the designed pocket, with an accuracy of only 60.8%. As a final test, we asked how our model performed on antibodies–specifically to identify antibodies that bind proteins or the glycans of glycoproteins. Of the 50 tested antibody structures, PiCAP achieved a 79% TNR and 33% TPR for a BACC of 56%. The antibodies and designed nonbinders are proteins hypervariably mutated at the binding site for specificity, which has the poorest performance of PiCAP, whereas PiCAP performs encouragingly on more evolutionarily and biologically defined proteins. We provide PiCAP and CAPSIF2 for open use on ROSIE [SERVER REFERENCE].

### PiCAP Agrees with LectomeXplore and Experimental Evidence.

Our NoCAP dataset for training and testing PiCAP comprises *experimentally* solved structures; therefore, we decided to investigate how our model performs on two datasets of *computationally predicted* structures. The first dataset is LectomeXplore published by Bonnardel et al. which identifies likely lectins across 37,794 organisms using a hidden Markov model (HMM) based on sequence and structural similarity (20). We also investigated the ganglioside interactome as published by Zhang et al. where they developed a high throughput assay to identify putative human proteins that interact with gangliosides (21). Both of these datasets have only sequence/UniProt gene IDs, therefore, for input into our algorithm, we used the predicted structures of the AF2 model proteomes (10), only retaining confident segments of the structure (pLDDT larger than 70).

#### LectomeXplore.

The most closely related work to PiCAP is LectomeXplore, which identified putative lectins through sequence and structure homology. Unlike LectomeXplore, PiCAP does not limit proteins to be only of the lectin superfamily. We compared the likelihood of all predicted LectomeXplore lectins (greater than 0.25 confidence) present in the AF2 reference proteomes of two model species, *Mus musculus* and *Homo sapiens*, finding the agreement between the available AF2 structures of LectomeXplore and PiCAP to be 100% (225 of 225) for *M. musculus* and 99.6% (229 of 230) for *H. sapiens*. Further, PiCAP has a 100% (109 of 109) agreement between the available AF2 structures on all confirmed human lectins from HumanLectome (24). These results suggest a strong true positive rate (TPR) of PiCAP on the simplest class of sugar binding proteins.

#### Ganglioside interactome.

Zhang et al. developed a high throughput method to identify proteins that interact with gangliosides. They created ganglioside probes with photoaffinity tags that covalently linked the probe to nearby proteins, and then they used mass spectroscopy and statistical methods to identify those proteins. They used six different probes in two different cell lines (A431 and SH-SY5Y), and for a total of nine experiments; we filtered the putative proteins by experiment. We selected the top 250 proteins above background from each experiment and removed CRAPome proteins (25). This identified 873 unique proteins across all nine experiments (21). As a high throughput method, and the first and largest of its kind, the error rates of their method have yet to be explored and cross-validated across other experimental methods. We therefore will use PiCAP to investigate the putative proteins of the ganglioside interactome work.

**Fig. 3.**
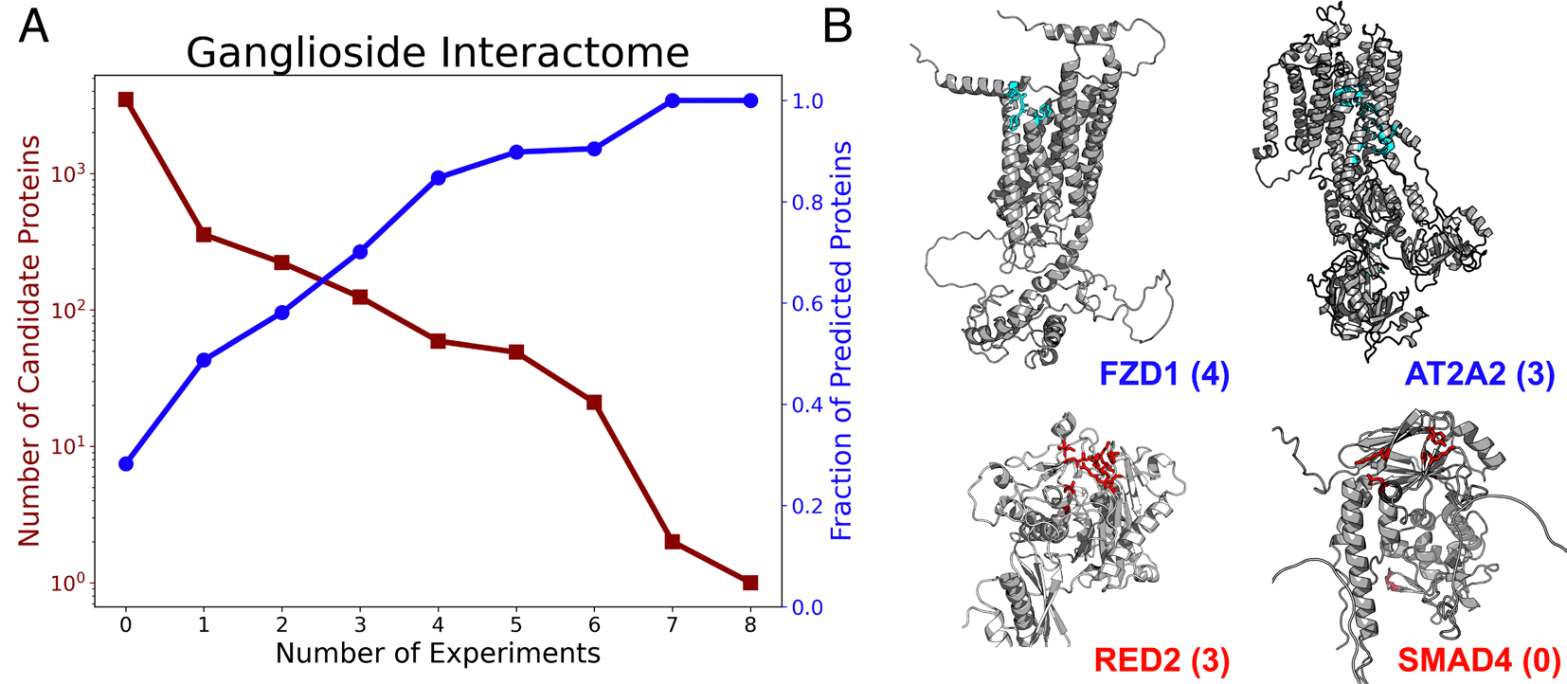
PiCAP validation against computational datasets. (*A*) Plot of Zhang et al. identified proteins across nine experiments alongside the fraction of proteins in each bin predicted as a carbohydrate binder by PiCAP. (*B*) PiCAP and CAPSIF2 predictions of selected ganglioside interactome protein structures from AF2 model human organism proteome. The *Top* row (blue) indicates proteins predicted as carbohydrate binders by PiCAP (FZD1, UniProt: Q9ULW2 and AT2A2, UniProt: P16615) and *Bottom* row (red) as proteins predicted as nonbinders (RED2, UniProt: Q9NS39 and SMAD4, UniProt: Q13485) by PiCAP with the number of experiments the protein was identified by in parentheses. Highlighted residues in cyan (*Top* column) and red (*Bottom* column) are the predicted binding regions by CAPSIF2.

Of the 873 identified candidate ganglioside binding proteins, we were able to identify 848 proteins in the AF2 reference human proteome. PiCAP predicts 506 (60%) of these proteins as carbohydrate binders. Further, PiCAP also predicts that 988 of 3,500 putative nonganglioside binding proteins (28%) as likely carbohydrate binders. Although these numbers at first suggest a substantial disagreement between our works, we see a strong positive increase in the fraction of proteins predicted as carbohydrate binders compared to the number of experiments that identified a binding protein (Fig. 3*A*).

To explore the agreement and disagreement between our experiments, we selected four representative proteins: Frizzled-1 (Entry Name: FZD1; UniProt: Q9ULW2), ATPase sarcoplasmic/endoplasmic reticulum Ca2+ transporting 2 (AT2A2; P16615), Double-stranded RNA-specific editase B2 (RED2; Q9NS39), and mothers against decapentaplegic homolog 4 (SMAD4; Q13485). FZD1 is involved in the Wnt signaling pathway and was identified by four Zhang et al. experiments; PiCAP predicts FZD1 as a carbohydrate binder, and FZD1 was a subject of close scrutiny in the ganglioside interactome work (21). ATP2A2 is an intracellular calcium/ATP pump and was identified by three experiments, and predicted as a carbohydrate binding protein by PiCAP. ATP2A2 has a specific role in ATP-mediated transport of calcium ions and likely little specific affinity for carbohydrates, let alone gangliosides (26). RED2 is an enzyme that converts adenosine to inosine in pre-mRNA and was identified by three experiments (27). PiCAP disagrees with the experimental results and predicts RED2 as a nonbinder, which could indicate a potential error in the experimental evidence. Finally, SMAD4 is a transcription factor (28), which was identified to not interact with gangliosides in all experiments, where PiCAP agrees and predicts the protein as a carbohydrate nonbinder.

### PiCAP and CAPSIF2 Can Predict Putative Proteome Scale Interactomes.

**Fig. 4.**
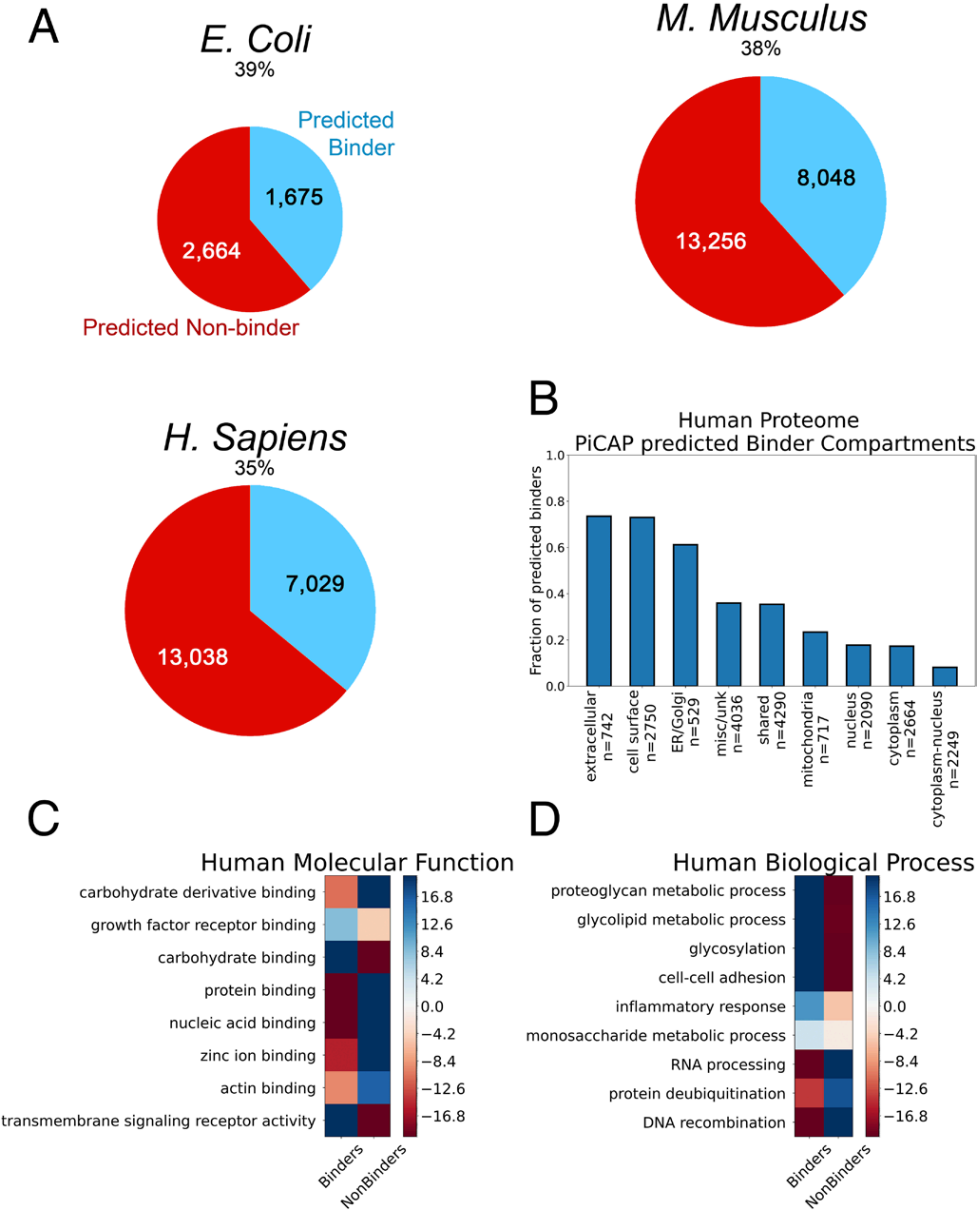
PiCAP predictions of proteomes. (*A*) Comparison of the fraction of proteins predicted as carbohydrate binders by PiCAP across three proteomes. (*B*) Cellular components of human proteome predicted carbohydrate binding and nonbinding proteins (*SI Appendix*, Table S3). Human proteome statistical tests showing the −log_10_ of the false discovery rate (FDR) and overrepresentation (blue) and underrepresentation (red) for select (*C*) molecular functions and (*D*) biological processes. FDR measures the expected proportion of false positives among the list of putative carbohydrate binders and nonbinders.

With PiCAP validated to an acceptable level, we sought to understand the protein–carbohydrate interactome with greater breadth than studied before. We chose three model organisms from the AF2 proteome datasets (10), *Escherichia coli*, *M. musculus,* and *H. sapiens*. Of the 4,363 proteins in the AF2 *E. coli* strain K12 proteome (UP000000625), PiCAP yielded predictions on 4,339 accessible proteins and predicted 1,677 (39%) proteins as carbohydrate binders. Of the 21,615 proteins in the AF2 *M. musculus* proteome (UP000000589), PiCAP yielded predictions on 21,304 proteins and predicts 8,177 (38%) proteins as carbohydrate binders. Of the 20,650 proteins in the AF2 *H. sapiens* proteome (UP000005640), PiCAP yielded predictions on 20,067 proteins and predicts 7,029 (35%) proteins as carbohydrate binders (Fig. 4*A*). We further provide the results of three additional model species: *Drosophila*, *Caenorhabditis elegans*, and *Saccharomyces cerevisiae* in the supplemental information (29).

A remarkable organism for studies of glycans and glycan recognition is *C. elegans* (30). In the genomic database searched (supplemental information), there are 227 proteins with C-type lectin domains, 23 galectins (twice as many as humans) and 274 glycosyltransferases among 19,227 protein-encoding genes. Using a PiCAP probability cutoff of 0.9, 33% of all *C. elegans* proteins were classified as binding carbohydrates. For C-type lectins, 97% fell into this category, for galectins 100%, and for glycosyltransferases 86%. For comparison, only 3% of *C. elegans* ribonucleoproteins fell into this category.

#### Human proteome.

The primary proteome we analyzed was the AF2 human proteome (UP000005640), which contains 20,650 unique proteins with substantial resolution. PiCAP predicted 7,029, or 34%, of proteins to bind to carbohydrates. For comparison, the total number of lectins identified by LectomeXplore is 230, or 1.1% of the UniProt reference proteome (20), and the number known by CAZY is 349, or 1.7% (19). In contrast, the number of proteins experimentally identified as likely to bind gangliosides, a unique glycan family, is 873, or 4.2% (21). To reconcile the differences between our work and the work of many others, we analyzed the subcellular localization, molecular functions, and biological processes of predicted binding and nonbinding proteins.

We investigated the subcellular compartments wherein PiCAP predicted carbohydrate binding proteins and nonbinders reside based on Gene Ontology (GO) terms (Fig. 4*B* and *SI Appendix*, Table S3). The compartments with the highest fraction of sugar-binding proteins are extracellular (75%), cell surface (75%), and ER/Golgi (50%), which aligns with these being subcellular compartments involved in intercellular communication. The regions mostly devoid of carbohydrates and glycans are the nucleus and cytoplasm; PiCAP predicts 85 to 97% of these proteins as non-carbohydrate-binding proteins. To further investigate the binding profiles of PiCAP, we queried cofactor binding. A significant portion of carbohydrate-binding proteins depend on a cofactor such as calcium in C-type lectins (2), whereas zinc is more dominantly oriented as a DNA/RNA binding cofactor (31). PiCAP predicts 58% of calcium-binding proteins and 22% of zinc-binding proteins as carbohydrate binding proteins, indicating that PiCAP does not conflate cofactor binding for carbohydrate binding (*SI Appendix*, Fig. S1). Additionally, PiCAP predicts 94% of human GO associated carbohydrate binding proteins as binders and 91.5% of GO associated DNA/RNA binding proteins as nonbinders, indicating an overall ~93% accuracy, which agrees with our NoCAP dataset evaluation (*SI Appendix*, Figs. S1 and S5).

To discern higher specificity from the AF2 human proteome predicted by PiCAP, we selected representative GO terms with PANTHER (32, 33). Using the false discovery rate (FDR) for human proteome related molecular functions, we find PiCAP-predicted binding proteins are significantly overrepresented to act in growth factor receptor binding, transmembrane signaling, and unsurprisingly, carbohydrate binding (Fig. 4*C*). Additionally, PiCAP-predicted binders are highly underrepresented for carbohydrate derivative binding but also protein binding, nucleic acid binding, zinc ion binding, and actin binding. Next, we analyzed the biological processes of human PiCAP predicted carbohydrate binding proteins, finding them overrepresented in proteoglycan and glycolipid metabolic processes, cell–cell adhesion, inflammation response, monosaccharide metabolic process, and unsurprisingly glycosylation (Fig. 4*D*). Comparatively, we found that carbohydrate binding proteins are underrepresented in RNA processing, protein deubiquitinization, and DNA recombination cellular processes. Analysis for *E. coli* strain K12 and *M. musculus* AF2 proteomes is provided in the supporting information (*SI Appendix*, Figs. S2–S6), showing similar predictions.

## Discussion

We have demonstrated 1) an updated protein–carbohydrate site identifier CAPSIF2 that outcompetes all current models on a generalized dataset and 2) a model named PiCAP that predicts *whether* a protein binds to carbohydrates or not. We validate our models against other models and datasets and applied to proteome scale analysis to garner more information about the protein–carbohydrate interactome.

CAPSIF2 boasts modest improvements in prediction accuracy on the original CAPSIF/TS90 dataset compared to CAPSIF:G and CAPSIF:V, but it underperforms PesTo-Carbs. CAPSIF2 however excels the most at a larger dataset containing ~1 k structures with substantially larger sequence variability, outcompeting all tested models. CAPSIF2 leverages a graph neural network operating on residues, using the same foundational approach as CAPSIF:G, while CAPSIF:V used a 3D voxelized CNN approach. PesTo-Carbs also leverages a graph neural network approach; however, it operates at an atom-wise level and only pools to the residue level late in the architecture. These graph architectures however have a similar level of parameters, where CAPSIF:G has 236 K parameters, CAPSIF2 has 1.6 M parameters, and PesTo-Carbs has 1.1 M parameters; while CAPSIF:V has substantially more with 102 M parameters.

We believe that the differences in performance are primarily not attributable to the architectures themselves, but rather the datasets. All models perform in a Matthews correlation coefficient (MCC) range from 0.55 to 0.63; thus, we attribute the largest differences to the stochastic training of these models and the slight variations in architectures. Structural protein–carbohydrate datasets are limited currently by the size of the PDB, as these interactions must be strong and stable to observe with experimental methods, where in physiology these interactions are often guided by avidity over affinity and/or enzymatic activity on the carbohydrates themselves. We believe larger datasets are only one part to improving these models, but improving the datasets with manual interrogation of all structures and with the identification of continuous biophysical pockets is necessary to improve the models’ performance.

To improve carbohydrate–protein structural datasets and improve our general biological understanding of the carbohydrate–protein interactome, we created PiCAP. PiCAP is a model that predicts the protein–carbohydrate interactome—carbohydrate binding of proteins independent of family/function—whether it be a cell surface protein for adhesion and communication or for metabolic enzymatics. The dataset we used to train PiCAP primarily separates known carbohydrate binders and proteins that are unlikely to bind to carbohydrates physiologically inside the cell—ranging from small molecule binders to cytoskeletal components. Although this approach is imperfect, it is the first attempt of this kind and, while limited by the underlying skewed PDB species distribution toward soluble proteins from human and simple prokarya, it still leverages a generalizable biophysical intuition of the cellular systems. Further augmentation of the NoCAP dataset could improve on the breadth of the training data by using sequence databases in conjunction with structure predictions such as AF3 (9) or Boltz (34). Additional positive sugar binders can come from CAZY (19, 35) which contains 5 M+ enzymes. Additional negatives can be identified across many species using specific GO terms with known presence in the cytoplasm, nucleus, or nuclear membrane.

Ultimately PiCAP achieves 89.6% accuracy on the experimental NoCAP dataset with 1.8 M parameters. PiCAP predicts most subsets of the test set with equivalent accuracy (designed lysozymes, cytoskeletal proteins, flippases, and fatty acyl binding proteins); however, it proves notably worse on designed nonbinders and antibodies. The designed nonbinders were created using Rosetta, where the binding pocket itself was designed but the remainder of the protein remained untouched (36). These designs were labeled as nonbinders by positive Rosetta binding energy scores–and never experimentally expressed nor tested. In a similar vein, to bind carbohydrates, antibodies use their hypervariable regions which are local regions that undergo somatic hypermutation. Our input to the protein is ESM2 embeddings, which uses full sequence context to extract a large 1,280-dimensional embedding of each residue. As the ESM2 model is only trained and tested on biological proteins, the signal specificity of the binding pocket sequence of the designed nonbinders may be masked by its more evolutionarily conserved residue, leading PiCAP to predict these nonbiological proteins as carbohydrate binders. PiCAP studies protein sequence and structural information together, indicating PiCAP as a strong candidate for proteome wide studies of protein–carbohydrate interactions.

Since PiCAP performed well on NoCAP data, we sought to validate the model against other methods that predicted carbohydrate binders: the ganglioside interactome and LectomeXplore. While we saw only 60% of proteins in the ganglioside interactome as positive, after closely evaluating a subset of the data, we reconciled the difference with the error of the high throughput experimental method. Although PiCAP appears to disagree with a good fraction of the ganglioside dataset, it has a strong linear relationship with the high throughput experiments. The more experiments that identified a protein, the higher likelihood that PiCAP predicted the protein as a carbohydrate binder. We further observe strong agreement between PiCAP (1.8 M parameters) and LectomeXplore (a sequence-based HMM), with 99%+ agreement across all tested model species, which is encouraging for further extrapolation of PiCAP on omics scales.

With experimental and computational validation, we then leveraged PiCAP against the AF2 proteome datasets. PiCAP predicts 35~40% of all proteins in three biological model species to be carbohydrate binding proteins–the highest prediction to date. As carbohydrates are ubiquitous across all species as intermediaries of energy storage and extracellular communication, it is unsurprising that a high fraction of proteins bind carbohydrates. We find that PiCAP predominantly identifies ~70% of extracellular and cell surface interact with carbohydrates, indicating potential localization and multiple binding modes of many extracellular proteins. PiCAP results can be further validated by proteomic evaluation by experiments such as the pull-downs from Zhang et al. (21) or liquid glycan arrays (37, 38). The computational predictions can help elucidate more functionality of proteins and provide a larger context to their roles inside the cell and the suggestion of more protein moonlighting than previously understood.

Although carbohydrates play critical biophysical roles across virtually all cellular functions, the full extent of protein–carbohydrate interactions remains poorly characterized. Our work expands to all proteins/carbohydrates in an agnostic manner that abstains from any limits on protein family or carbohydrate species. Although this one-size-fits-all approach cannot distinguish metabolic from cell surface carbohydrates; this work suggests most extracellular proteins bind carbohydrates in some capacity and lays the foundation for future research to create a tailored approach to identify which carbohydrates these proteins bind.

An inherent limitation of the current method is the definition of “carbohydrate,” a term that includes sugars, polysaccharides, and a wide variety of glycans (39, 40). Carbohydrates include parent polyhydroxy aldehydes and ketones, hundreds of structurally related monosaccharides and derivatives thereof (natural and synthetic), and abundant covalent combinations of these. No effort is made here to define or represent the breadth of carbohydrates. Instead, the term carbohydrate here is limited to the structures captured in the datasets on which the machine learning is based (*Materials and Methods*). These are nearly all pyranoses and overrepresented by animal glycans constructed primarily of just nine monosaccharides (41). Future iterations of this approach may benefit from expanding the focus to broader sets of carbohydrates or narrowing to specific carbohydrate subsets.

We released the results of CAPSIF2 and PiCAP of six model system proteomes for all proteins for open-source scientific use. Additional steps can now be taken for the ultimate goals to design proteins to carbohydrate and glycoprotein targets for therapeutic purposes. First, we encourage the expansion of this work or LectinOracle (42) or GlyNet (43) to predict carbohydrate species to all carbohydrate binding proteins. One simple step would be to predict whether proteins bind to just a specific species of carbohydrate–such as chitins or sialic acids. Another step would a high throughput computational docking of those carbohydrate species to the identified proteins, using CAPSIF2 or PesTo-Carbs (15) or DeepGlycanSite (16) to identify an initial hypothesis to feed GlycanDock (44), or directly de novo with programs like DiffDock (11), RosettaFold-All Atom (RF-AA) (45), AlphaFold3 (9), or Boltz-1 (34) as explored in Canner et al. (46).

In addition, all these methods leverage deep learning techniques. Deep learning methods require multitudes of data, and although we were able to demonstrate impressive results on low accuracy/messy data, we believe a clean dataset is integral and necessary for the future of this field. A better annotated set of proteins that do not bind carbohydrates would be helpful, as well as all structural proteins to have all ligands together, where currently there is a high redundancy in protein structures with slightly different ligands or crystallization techniques, which reduce the accuracy of the test metrics in comparing CAPSIF2 and PesTo-Carbs.

Our experiments list the binding probability of approximately 85,000 proteins across six species. Validation to date is based on the accuracy of these predictions in reflecting previously confirmed carbohydrate-binding proteins and noncarbohydrate binders. Most predicted carbohydrate binding interactions, however, have limited to no experimental evidence indicating their carbohydrate binding ability. To assess the binding capacity of uncharacterized *E. coli* predicted proteins (*SI Appendix*, Fig. S6) and ganglioside binding proteins (Fig. 3), we performed a de novo spot-check by evaluating the protein structure and predicted binding interface. Although these checks provide some insight, further validation will require prospective evidence of direct carbohydrate binding by individual proteins. The ability to screen for protein–carbohydrate binding is currently limited due to the intrinsic nature of these interactions. Even when the specific glycan ligand is known, the affinities of protein–carbohydrate binding sites are typically low, with *K_D_* values in the micromolar range (47) and binding half-lives of a few seconds. Nature resolves this, as needed, using multivalent presentations of the binding proteins and target glycans. Multivalent interactions geometrically enhance binding affinity (48). For example, human Siglec-8 (PiCAP probability 0.993) has a site affinity for its primary target glycan of ~300 µM but multivalent binding affinity to its native ligand of 60 pM (49, 50). Protein binding to multivalent glycan arrays can be performed, but typically with native or artificially constructed multivalent proteins. Another limitation is that arrays are limited to a subset of printed glycan structures (47). Site binding of glycans in the micromolar range can be detected and quantified using isothermal titration calorimetry (49), but only when the binding glycan structure is known. In the case of Siglec-8, for example, the optimal glycan ligand is a relatively rare di-sulfated trisaccharide that would not be predicted intuitively. Additional methods to enhance detection of protein binding to glycan libraries are under investigation (48).

To further test our predictions, experiments, such as those done by Zhang et al. (21) or selective exo-enzymatic labeling (SEEL) glyco-engineering high throughput methods (51) are critical. These experimental techniques could further demonstrate a larger wealth of carbohydrate binding proteins, alongside their specificity, allowing for further annotation of the genome on a large scale.

In total, we present a framework to predict the protein–carbohydrate interactome across any species. Taking a carbohydrate agnostic approach, categorizing glycans and metabolic glucose together, PiCAP accurately predicts evolutionarily conserved proteins as carbohydrate binding proteins with approximately 90% accuracy (Table 3 and *SI Appendix*, Figs. S1 and S5). PiCAP’s predictions align with established biophysical principles, indicating that carbohydrate binding is largely absent from the cytoplasm and nucleus and approximately 75% of all cell surface and extracellular proteins bind carbohydrates (Fig. 4). This suggests that a majority of membrane, surface, and extracellular proteins may predominantly interact with glycans for localization and binding, rather than entirely relying on protein–protein specific interactions. These findings highlight the potential of PiCAP to not only accelerate glycoproteomic research but also refine our understanding of protein function in the broader context of cellular communication and molecular recognition.

## Materials and Methods

### Dataset.

From a machine learning perspective, the definition of carbohydrate depends on the data input. We selected carbohydrate-binding proteins by combining multiple datasets including carbohydrate binding antibodies from SAbDab, (52) all experimentally solved proteins from UniLectin (17) (with and without bound carbohydrates), the CAPSIF dataset, (14) and most notably, the DIONYSUS dataset (18), which was filtered for only carbohydrate containing complexes. Further, we included the computationally designed and experimentally viable lysozymes from ProGen, (23) with structures predicted by the Colab distribution of AlphaFold2 (53). The identity of carbohydrates represented in the carbohydrate-binding structures in Dionysus, UniLectin, and SabDAb are provided in the database statistics. In the Dionysus’ core dataset (18) 94% of the structures are bound to pyranoses, with glucose (57%), galactose (12%) and mannose (9%), xylose (7%), fucose (3%), and sialic acid (3%) most abundant. In UniLectin (17) nearly all the structures bind pyranoses, with galactose (31%), GlcNAc (16%), glucose (15%), mannose (12%), fucose (9%), sialic acid (9%), and N-acetylgalactosamine (7%) well represented. In SabDAb (52), the subset of antibodies that bind carbohydrates predominantly recognize pyranoses (96%) including GlcNAc (25%), the bacterial carbohydrate KDO (21%), mannose (16%), glucose (9%), and galactose (9%). These database statistics indicate that the structures sampled as carbohydrate-binding in the databases largely represent those that bind common pyranose structures expected to share the physical characteristics most important to glycan binding including CH-π central stacking surrounded by hydrogen bonding constituents (5), among others.

There are several datasets of protein–carbohydrate interactions; however, there is no dataset of proteins that do not bind to carbohydrates, so we constructed one (Table 1). In the creation of such dataset, an intrinsic difficulty is that it is not possible to prove that a protein does not bind to a carbohydrate of any kind; therefore, we selected proteins that biophysically have low likelihood to bind to carbohydrates due to their function or location inside the cell. The experimentally solved proteins selected were primarily chosen as small molecule binding proteins, DNA binding proteins, nuclear pore complex proteins, serine proteases, cytoskeletal proteins, aminotransferases, flippases, fatty acid binding proteins, selected antibodies (antibodies), and ribosomal proteins. In addition to these proteins, Luo et al. computationally constructed a dataset of carbohydrate nonbinder proteins with the Rosetta software (36).

Small molecule data constitutes the largest portion of the nonbinders (~18 k pdbs), as we used the PDB-Bind 2020 dataset (54). Some proteins in the PDB-Bind dataset contain carbohydrates as the ligand, in which case we identified those ligands using PyRosetta (55) and removed them from the nonbinder dataset and added them to the binder dataset. Antibodies were selected using the SAbDab dataset by finding all proteins that were bound to proteins or nucleic acids and further filtering to structures not containing any carbohydrates in the structure nor an NX(S/T) motif in the antigen (52). Ribosomal proteins were selected from the bacterial ribosome structure (56). The remainder of protein structures were selected by inspection from the RCSB PDB (57).

After combining the datasets and adjusting for duplicate PDBs across different datasets, the final NoCAP dataset contains 30,429 total unique protein structures. Of these structures, 9,509 bind to carbohydrates, with 6,262 having an experimentally bound carbohydrate. Of the 20,920 non carbohydrate binders, 17,191 have an experimentally resolved small molecule bound to it, leaving 3,729 as nonbinders. To encourage generalizability to minor errors in structure predictions, we also reconstructed the 12,021 shortest sequence proteins of the 30,429 with the Colab implementation of AlphaFold 2 (53), where we only kept the 11,042 of predicted structures with a pLDDT greater than 80.

### Preprocessing.

With our dataset, we desired to leverage both sequence and structural information to predict carbohydrate-binding capabilities of proteins. Family information of sequence similarity can strongly indicate carbohydrate binding capabilities, while structural motifs can be present across protein families for carbohydrate binding, and we desire our method to identify both. We extracted the sequence and the Cβ positions of all protein residues (Cα for glycine) using PyRosetta (55). Next, we used ESM2 (13) to provide a high-dimensional sequence embedding for each protein residue of each protein chain. We labeled protein residues that were within 4.2 Å of a noncovalently bound carbohydrate (or small molecule) as a binding residue.

Most previous work has used single protein chains for protein–carbohydrate predictions (14, 15); however, many proteins only exist in the context of multiple chains. For this reason, we preprocessed all protein structures with all chains in the PDB file, except the initial CAPSIF dataset and antibodies. To limit the redundancy of the training set, we used MMseqs to cluster protein sequences by 60% sequence identity into distinct clusters for training/testing (58). We then split the clusters into an 80/5/15 train/validation/test, maintaining the same proteins from CAPSIF remain in the same dataset distribution. This left 24,576 structures in training, 1,473 structures for validation, and 4,380 structures for testing.

### Secondary Validation Set.

We have a primary dataset of carbohydrate–protein binding; however, we need to demonstrate the ability of PiCAP to predict outside of the crystally solved structures. To do this, we gathered all UniProt (59) accession codes from Zhang et al. (21). and LectomeXplore (20) and matched them to the AF2 publicly accessible organism proteomes. This captured 848 of 878 (97%) of putative ganglioside binding proteins and 3,400 of 4,335 (78%) of nonganglioside binding proteins. LectomeXplore uses sequence and structural protein information, alongside infectious pathogens that affect these species, and lists all reference sequences and structures (UniProt, ensembl, NCBI, RCSB, etc.) with severe redundancy. Therefore, for a direct quantitative comparison, we therefore used only those that existed singly as UniProt values inside the reference proteomes of AF2. We used the confidence metric of 0.25 for identification of lectins, which yielded 230 human proteins and 225 mouse proteins.

For our proteome analysis, we used the AF2 publicly accessible organism proteomes (10). AF2 generates structures with an internal confidence metric called pLDDT, where low confidence regions will have pLDDTs under 70. We therefore performed analysis and studies on AF2 protein regions with high confidence, or residues with greater than 70 pLDDT, independent of structural continuity. We applied the analysis to the following model organisms: **E. coli*, M. musculus,* and *H. sapiens*. We further provide the results of the full-length sequence, independent of pLDDT in Supplemental information alongside the results of three other model organisms: *C. elegans*, *Drosophila melanogaster*, and *S. cerevesia*.

### Architectures.

We fed the residue coordinates and sequence embeddings into the CAPSIF2 and PiCAP architectures (Fig. 1*A*) into the main model block, which uses a message passing equivariant graph neural network (EGNN) of equivariant graph convolutional layers (EGCL) (60). Each layer sums the outputs of a multilayer perceptron (MLP) that inputs the features of the central node and the features of all of its neighboring nodes and the edge attributes of the neighbors. Following Ingraham et al. (61), the edge attributes are a radial basis function (RBF) of the distance, the orientation, and direction of the neighboring residues.

**Fig. 5.**
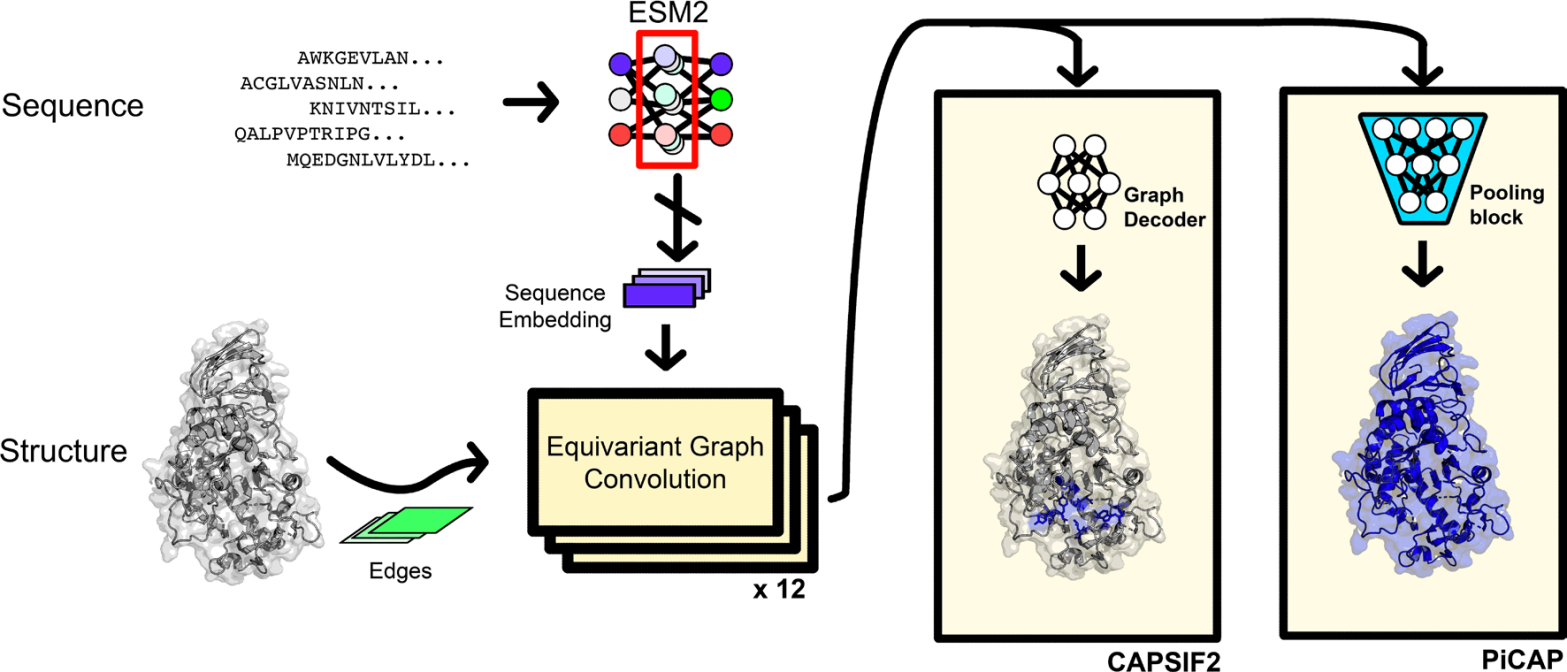
Architectures of CAPSIF2 and PiCAP.

CAPSIF2, a carbohydrate binding residue predictor, has 12 residual ECGLs with an embedding dimension of 128 (Fig. 5). The neighborhood context window is fixed at the 16 nearest neighbors. After the graph convolutions, each residue is passed to a two-layer dense decoder, finally outputting the carbohydrate-binding likelihood of each residue. CAPSIF2 contains 1,600,387 parameters.

PiCAP, a predictor of whether a protein binds to carbohydrates, has 12 total residual EGCLs with an embedding dimension of 128 and leverages an increasing neighborhood context window for information propagation, as inspired by PeSTo (62) and PeSTo-Carbs (15). The first three layers use the 10 nearest neighbors, layers 4 to 6 use the 20 nearest neighbors, layers 7 to 9 use 40 neighbors, and layers 10 to 12 use 60 neighbors (Fig. 5). The model specific block is a pooling block that uses an adaptive pool to truncate or slightly expand the size of the protein to a fixed length (150), where the model then uses two convolutional layers and three dense layers to predict the likelihood of a protein to bind to carbohydrates. PiCAP contains 1,798,895 parameters.

### Training.

We trained both models using two cycles: small molecule binding residue prediction and the model specific task (protein or residue level predictions). The first training cycle used the CAPSIF2 base architecture with randomized initial weights $∼N\left(0,0.02\right)$ for the residue level prediction. The model was trained for a maximum of 1,000 epochs, with training prematurely stopped once the validation loss did not decrease after 35 epochs. This training cycle had a learning rate of 2 × 10^−6^ and a weight decay of 10^−7^ with the Adam optimizer with the loss function *L* = 1 − *d*, where *d* is the Dice-Sorenson coefficient (also known as the F1 score) and a batch size of 1. To improve model generalization, each epoch sampled a single protein from every training cluster available from the small molecule dataset. The smallest 12,000 protein sequences were modeled structurally with the colab distribution of AF2, (8, 53) and if the selected protein was available via AF2, we selected the crystal structure 40% of the time and the AF2 structure 60% of the time.

For the second training cycle, CAPSIF2 used the same architecture as the first training cycle and required no randomization. CAPSIF2 was trained only on proteins with experimentally determined carbohydrate binding sites with learning rate of 2 × 10^−5^ and weight decay of 10^−6^ with the Adam optimizer and the same loss function of *L* = 1 − *d*. Similar to the first training cycle, we randomly selected an available AF2 structure 60% of the time.

For the second training cycle, PiCAP used the weights where available from the first training iteration of CAPSIF2 and randomized weights for the model specific block ∼N(0,0.02). PiCAP was trained on the entire training set for binary classification with a learning rate of 2 × 10^−5^ and weight decay of 10^−6^ with the Adam optimizer and binary cross entropy (BCE) loss function. Similar to the first training cycle, we randomly used an available AF2 structure 60% of the time.

## Supplementary Material

Appendix 01 (PDF)

## Data Availability

Data, code, and datasets are available at Github, where CAPSIF2 and PiCAP can be run at: https://github.com/Graylab/picap (29). We further provide a webserver on ROSIE where CAPSIF2 and PiCAP can additionally be run: https://r2.graylab.jhu.edu/apps/index (63) data have been deposited in Github. Other data are included in the article and/or *SI Appendix*.
