# Supplementary material for: Predictions from deep learning propose substantial protein–carbohydrate interplay
Source: Proc Natl Acad Sci U S A. 2026 May 18;123(21):e2523342123. doi: 10.1073/pnas.2523342123 (PMC13213957; doi:10.1073/pnas.2523342123)
Supplement: Supplementary file 1 — Appendix 01 (PDF) [file pnas.2523342123.sapp.pdf]

Supporting Information

Predictions from Deep Learning Propose Substantial Protein-Carbohydrate Interplay

Samuel W. Canner[1], Ronald L. Schnaar[2,3], Jeffrey J. Gray[1,4]
[1]Program in Molecular Biophysics, Johns Hopkins University, Baltimore, MD, United States
[2]Department of Pharmacology and Molecular Sciences, Johns Hopkins University School of Medicine, Baltimore, Maryland, United States
[3]Department of Neuroscience, Johns Hopkins University School of Medicine, Baltimore, Maryland, United States
[4]Department of Chemical and Biomolecular Engineering, Johns Hopkins University, Baltimore, MD, United States
* Correspondence: Jeffrey J. Gray, jgray@jhu.edu

Dataset Description

We provide Supplemental File 1 as an Excel document (xlsx). This Excel document contains all prediction information of PiCAP and CAPSIF2 on the AlphaFold 2 proteomic data[1]. Since PiCAP can produce false negative binders, the separate predictions of CAPSIF2 in all cases may assist in hypotheses of known carbohydrate and small molecule binding proteins. In addition, we show predictions on all proteins in the dataset, even in cases with low pLDDT, although our analysis in the primary text only analyzes predictions of proteins with greater than an average of 70 pLDDT. In all sheets, we provide the following columns:

- UniProt Entry
- Common Gene Name (Entry_Name)
- Protein_name
- Gene Ontology terms
- PiCAP prediction on only residues with greater than 70 pLDDT
- CAPSIF2 predicted binding residues on residues with greater than 70 pLDDT

The PiCAP output is a probability value in the range from 0 to 1. In the main text we use the cutoff value of 0.23 to indicate that any protein with predicted probability greater than 0.23 is predicted as a carbohydrate binding protein. The PiCAP prediction may be used as a confidence metric, where the higher probabilities suggest more confidence PiCAP has that the model is a carbohydrate non-binder or binder, respectively.

Model Hyperparameterization

To optimize performance on a neural network, we assessed multiple hyperparameters in both models to achieve their performance. We focused primarily on the following hyperparameters: embedding dimension, *k*-nearest neighbors (knn), and number of layers. For simplicity, we treat each network as a series of four (4) blocks, composed of a certain number of layers where we vary knn per block.

CAPSIF2 parameterization

In our previous work on CAPSIF:G, we used one-hot encodings of amino acid type and biophysical properties with simple edge embeddings. To contain more information, in this work, we altered the node features to ESM2 embeddings and edges. With these input features, we then focused on the size and depths of the network.[2] A full account of all tested hyperparameters is listed below in Table S1. We selected CAPSIF2 as the model that performed the best on the DR test set, which was composed of 12 layers with a static number of k nearest neighbors of 16.

Table S1: Performance of various CAPSIF2 models on the Dionysus Residue (DR) test set. Dice and Matthews correlation coefficient (MCC) are as defined in the main text. Boldface indicates the best performance in each metric. Selected CAPSIF2 model is highlighted in yellow.

Layers per block	KNN per block	DR Dice	DR MCC	TS90 Dice
3	6,6,6,6	0.541	0.542	0.533
3	8,8,8,8	0.477	0.484	0.431
3	8,12,16,20	0.391	0.407	0.366
3	6,10,14,18	0.528	0.528	0.572
3	10,20,40,60	0.289	0.312	0.364
3	16,16,16,16	**0.573**	**0.574**	0.616
3	20,20,20,20	0.498	0.496	0.575
4	8,12,16,20	0.566	0.567	**0.639**
4	6,10,14,18	0.408	0.419	0.319
4	8,8,8,8	0.491	0.486	0.548
CAPSIF:V	N/A	0.226	0.202	0.608

PiCAP parameterization

We followed the same methodology as CAPSIF2 to identify the strongest performing PiCAP model parameters. Our hyperparameter search is provided below in Table S2. The decision on which model performed strongest was less straightforward than CAPSIF2, as all multiple models performed strongly across the NoCAP test set. We selected a model that performed well across most metrics placing just below the top of every other category to encourage generalizability, as some of the top performing models were prone to overfitting and unstable predictions. The chosen PiCAP model consisted of 12 layers, with the knn gradually increasing from 10 to 60 neighbors across the layers.

Table S2: Performance of various PiCAP models on the NoCAP test set. BACC is balanced accuracy. TPR is True Positive Rate TPR = TP / (TP + FP). TNR is True Negative Rate TNR = TN / (TN + FN).

Layers per block	KNN per block	cutoff	NoCAP BACC	NoCAP TPR	NoCAP TNR	Nonbinders TNR	Ribosome TNR	Holdout TNR
3	6,6,6,6	0.94	0.85	0.87	0.83	0.624	**1.0**	0.857
3	**8,8,8,8**	**0.33**	0.892	0.964	0.82	0.667	0.857	**0.929**
3	20,20,20,20	0.21	0.856	0.88	0.833	0.683	**1.0**	0.429
3	10,20,40,60	0.23	0.896	0.963	0.828	0.608	**1.0**	0.902
3	6,10,14,18	0.99	0.779	0.927	0.631	0.656	0.857	0.571
4	6,6,6,6	0.77	0.885	**0.976**	0.794	0.731	**1.0**	0.857
4	8,8,8,8	0.19	**0.897**	0.951	**0.842**	0.704	**1.0**	0.857
4	6,10,14,18	0.32	0.861	0.974	0.745	0.134	0.857	0.643
4	8,12,16,20	0.84	0.877	0.954	0.801	**0.785**	**1.0**	0.643

Proteomic Data

In Supplemental File 1, we provide a list of all proteins from six organisms. Here we list the overall metrics of the three organisms in Supplemental File 1 that were not discussed in the main text. PiCAP predicts that in *C. elegans* (nematode worm) 9,278 of the 19,227 proteins (48%) bind carbohydrates. PiCAP predicts that in *D. melanogaster* (fruit fly) 5,248 of the 13,351 proteins (39%) bind carbohydrates. PiCAP predicts that in S. *cerevisiae* (yeast) 1,749 of the 5,849 proteins (29%) bind carbohydrates. We do not provide any further analysis of these proteomes; however, provide a more in-depth analysis of PiCAP's predictive capabilities on the human proteome, and a similar analysis to the main text for the *M. musculus* and *E. coli* strain K12 proteomes below.

Cellular component analysis

To analyze the *E. coli* strain K12*, M. musculus,* and *H. sapiens* AF2 reference proteomes, we employed the use of Gene Ontology (GO) terms and PANTHER. For Figure 5B, we analyzed the GO terms representative of cellular compartments, we performed a limited search limited to Table S3, where any protein observed in multiple of the compartments (excluding just nucleus and cytoplasm) were placed in the "shared" compartment. PANTHER provided the statistical overrepresentation tests and false discovery rates (FDRs) of all cellular compartments, molecular functions, and cellular processes (when the FDR was less than 0.05).

Table S3: **Simplified cellular compartment GO Terms**

Compartment	GO Terms
Cell Surface	cell surface [GO:0009986]<br>plasma membrane [GO:0005886]<br>extracellular space [GO:0005615]<br>extracellular matrix [GO:0031012]
Cytoplasm	cytosol [GO:0005829]<br>cytoplasm [GO:0005737]
Nucleus	nucleus [GO:0005634]
Mitochondrion	mitochondrion [GO:0005739]
ER/Golgi	endoplasmic reticulum [GO:0005783]<br>Golgi apparatus [GO:0005794]

Supplemental Human Proteome Analysis

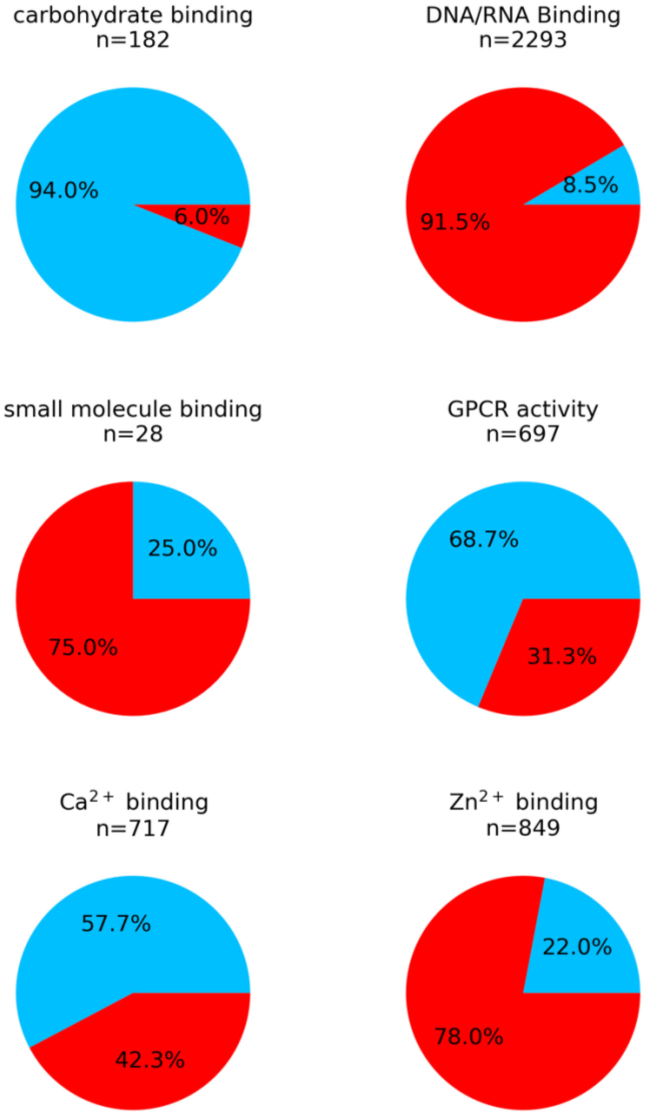

Figure S1: Human carbohydrate binding protein functionality. Percentage of proteins with known binding functions predicted as carbohydrate binding (blue) and non-carbohydrate binding (red) proteins for sets of proteins with Gene ontology terms for carbohydrate binding [GO:0030246], DNA and RNA binding [GO:0003677, 0003723], small molecule binding [GO:0036094], GPCR Activity (G protein-coupled receptor activity [GO:0004930]), Calcium binding (calcium ion binding [GO:0005509]), and (Bottom Right) Zinc binding (zinc ion binding [GO:0008270]).

To assess the overall accuracy of PiCAP, we identified the percentage of proteins with GO terms associated with carbohydrate binding, DNA/RNA binding, small molecule binding, G protein coupled receptor (GPCR) activity, calcium ion binding, and zinc ion binding (Figure S1). Of the 182 proteins with a GO term for carbohydrate binding in the human proteome, PiCAP

predicts 94% of these proteins as carbohydrate binding proteins, indicating a 6% false negative rate.

Here, we defined nucleic acids as non-carbohydrates, and PiCAP identifies 91.5% of DNA/RNA binding proteins as carbohydrate non-binders. PiCAP predicts 8.5% of DNA/RNA binding proteins as carbohydrate binding; where several nucleic acid binding proteins are known to bind carbohydrates, such as DNA polymerase I (such as in PDB 1NK4), so those predicted binders cannot be completely ignored as false positives. There are limited proteins with small molecule binding GO terms associated (28), and PiCAP predicts only 25% of these proteins to bind carbohydrates, which could include small molecules with hydrated ring structures, mimicking carbohydrate epitopes. Additionally, zinc is an ion commonly associated with nucleic acid binding with zinc finger motifs; however, zinc is established in other pathways like neuron excitability. PiCAP predicts 22% of known zinc ion binding proteins as carbohydrate binding proteins, and 78% as carbohydrate nonbinders.

We further assessed calcium ion binding, where calcium is ubiquitous across many cellular processes from muscular contraction to being a secondary ligand necessary for C-type lectin binding. In NoCAP, there are 2263 structures containing calcium, where 946 (41%) of those proteins bind carbohydrates. In the training set specifically, there are 1619 proteins that have calcium ions present and only 614 (38%) of those bind carbohydrates. On the human proteome, PiCAP predicts 58% of calcium binding proteins as carbohydrate binders and 42% as carbohydrate non-binders, indicating that PiCAP effectively distinguishes C-type lectins and calcium cofactor-carbohydrate binding proteins from other calcium binding proteins.

Finally, we investigated GPCRs, where proteins with GPCR activity are integral across many unrelated intercellular communication pathways, where PiCAP predicts 69% of proteins with GPCR activity as carbohydrate binding proteins, suggesting a wealth of agonists and antagonists being carbohydrates (or carbohydrate-like molecules with hydrated rings) may be critical in GPCR binding.

E. Coli Proteome analysis

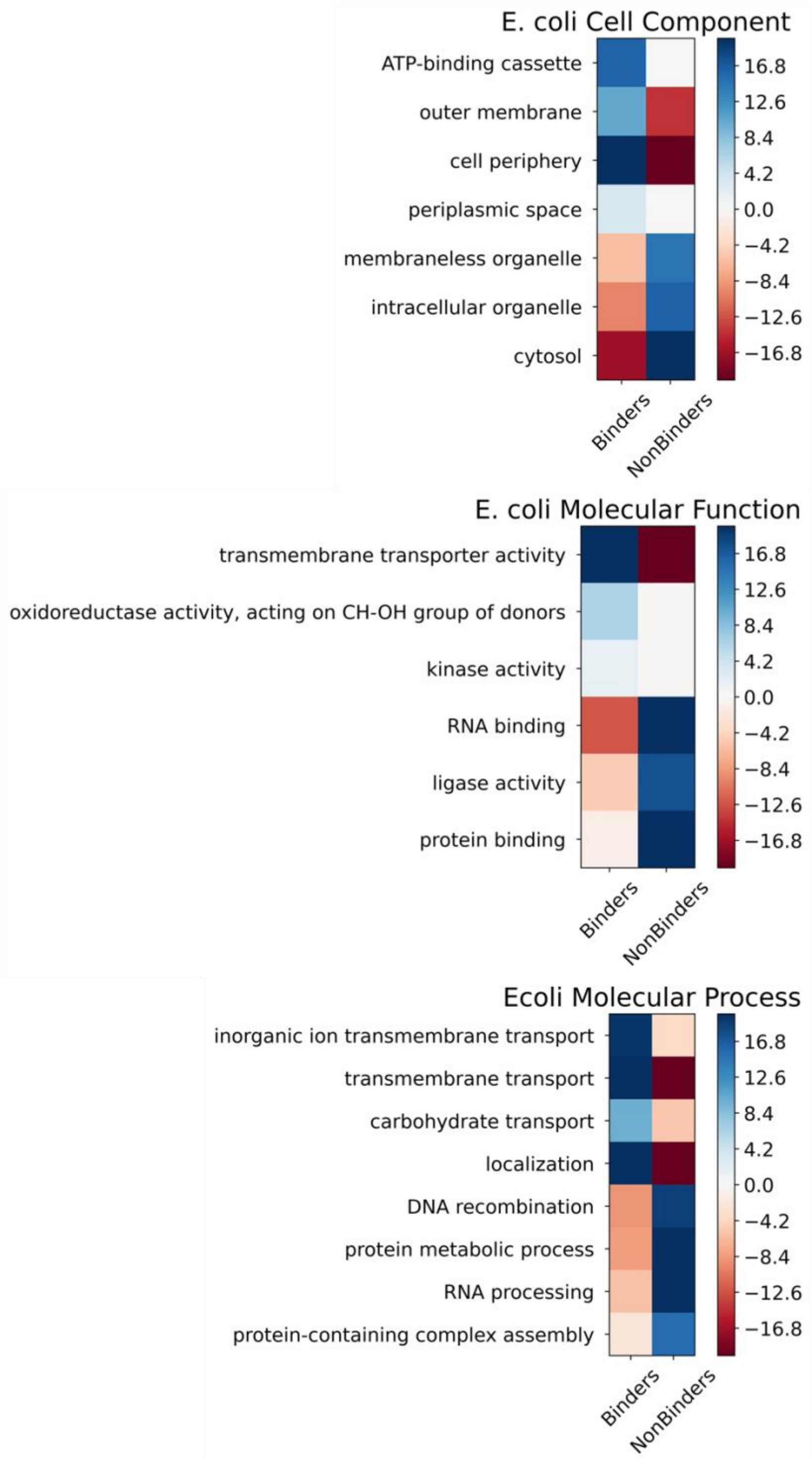

Figure S2: Statistical analysis of *E. coli* strain K12 PiCAP predicted carbohydrate binding and non-binding proteins. The false discovery rate (FDR) alongside the overrepresentation (blue) and underrepresentation (red) are shown for select cellular compartments, molecular functions, and cellular processes.

Mouse Proteome Analysis

Cellular Component

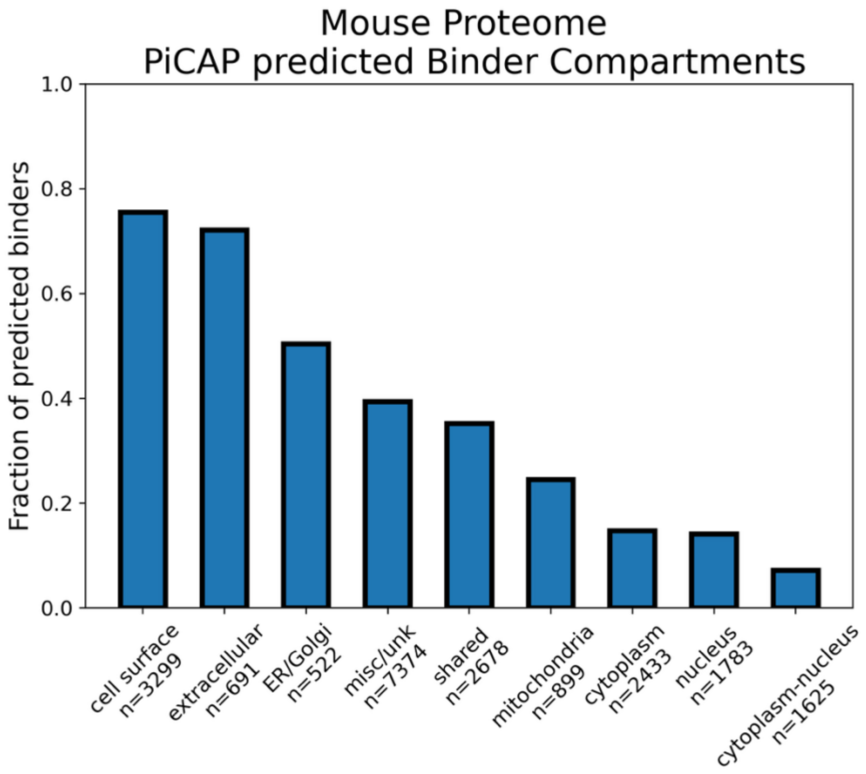

Figure S3: Cellular components of *M. musculus* proteome predicted carbohydrate binding and non-binding proteins according to Table S3.

Overrepresentation of protein processes and functions

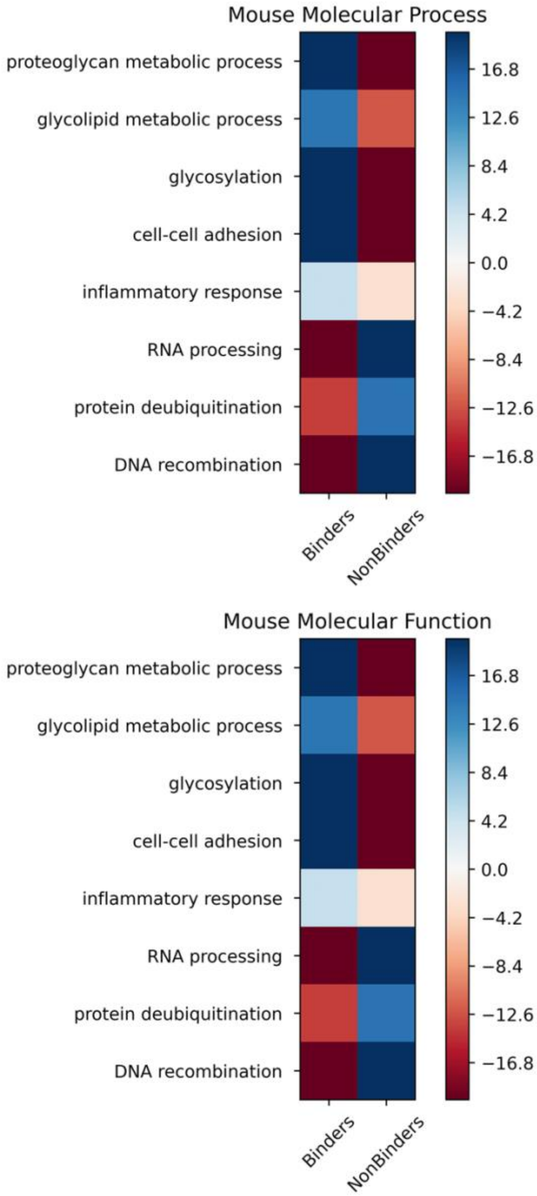

Figure S4: Statistical analysis of *M. musculus* PiCAP predicted carbohydrate binding and non-binding proteins. The FDR and overrepresentation (blue) and underrepresentation (red) are shown for select cellular functions and molecular processes.

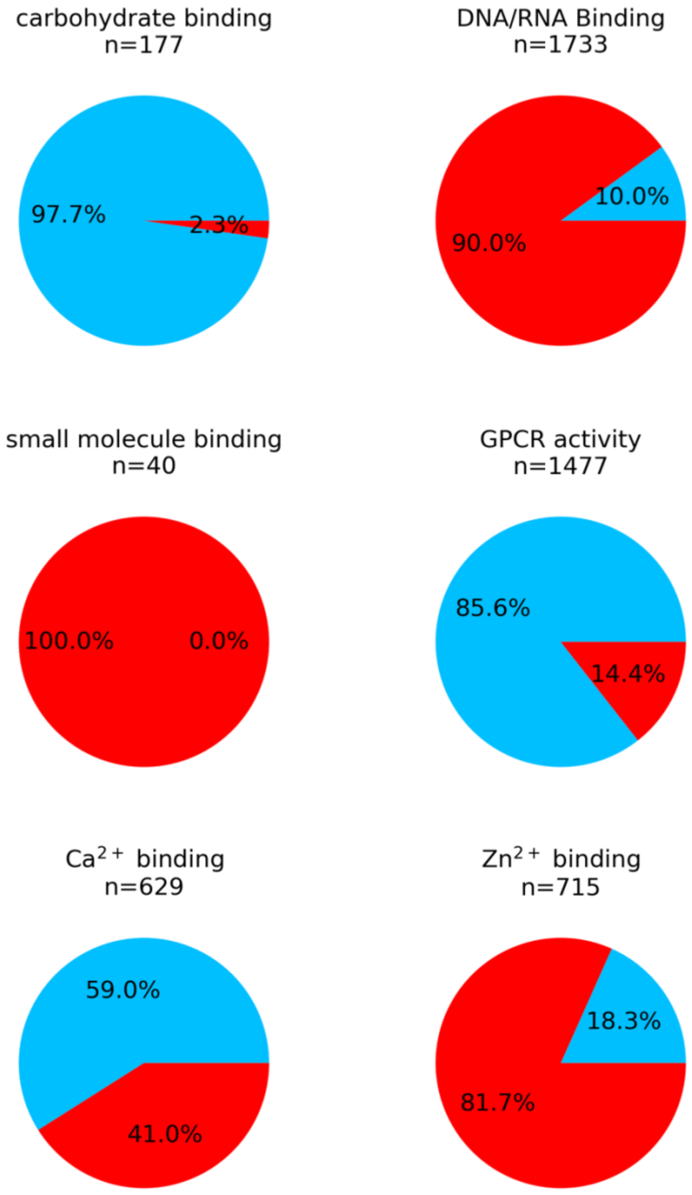

Figure S5: *M. musculus* carbohydrate binding protein functionality. Percentage of proteins with known binding functions predicted as carbohydrate binding (blue) and non-carbohydrate binding (red) proteins for Gene ontology terms for carbohydrate binding [GO:0030246], DNA and RNA binding [GO:0003677, 0003723], small molecule binding [GO:0036094], GPCR Activity (G protein-coupled receptor activity [GO:0004930]), Calcium binding (calcium ion binding [GO:0005509]), and (Bottom Right) Zinc binding (zinc ion binding [GO:0008270]).

E. Coli Uncharacterized Proteins

PiCAP is the first algorithm to predict whether a protein binds carbohydrates. We therefore used PiCAP to investigate potential carbohydrate binding functionality in uncharacterized proteins in *E. coli* strain K12. In this context, we define an uncharacterized protein as a one that lacks any

gene ontology terms (GO) in UniProt[3] and is annotated as an "uncharacterized protein" by EcoCyc (as of August 18, 2025).[4] Since carbohydrate binding is implicated in multicellular processes and proliferation, knockout of these specific genes of carbohydrate binding may not yield cell death or observable phenotypic changes in single-celled bacteria and archaea.

EcoCyc identifies 116 uncharacterized proteins that were also present in the AF2 proteome. PiCAP predicts that 42 (36%) of these proteins are likely carbohydrate-binding proteins. Three example proteins are shown in Figure S6: P28915 (YbfC), P37635 (YhiS), and P36682 (YacH). The AF2 model of YbfC adopts a β-sandwich fold, a canonical fold of lectins, with CAPSIF2 predicting residues on the β-sandwich. The AF2 model of YhiS contains two β-sheets potentially suitable for carbohydrate binding; however, CAPSIF2 does not predict any binding residues. The AF2 model of YacH primarily consists of α-helices and β-strands, with additional floating α-helices which may be part of a different domain; nevertheless, CAPSIF2 does not predict any residues to bind, and PiCAP only predicts a 72% likelihood of carbohydrate-binding. We hope other groups will test these hypotheses of binder and non-binder labels experimentally.

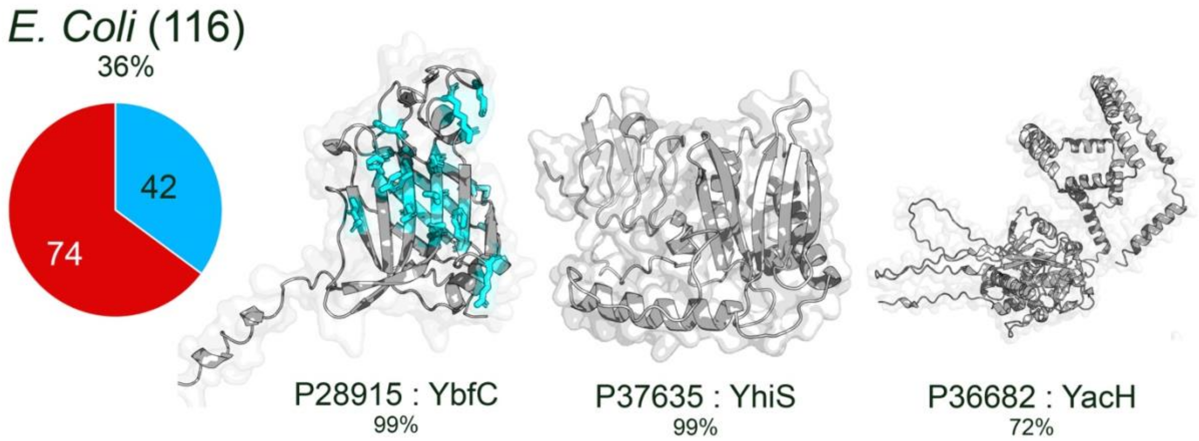

Figure S6: PiCAP predictions of *E. Coli* strain K12 uncharacterized proteins according to EcoCyc.[4] (Left) Comparison of the fraction of proteins predicted as carbohydrate binders by PiCAP across three proteomes. (Right) PiCAP and CAPSIF2 predictions of three selected *E. Coli* strain K12 AF2 predicted protein structures with PiCAP prediction confidence below.

References

1. Varadi, M. *et al.* AlphaFold Protein Structure Database in 2024: providing structure coverage for over 214 million protein sequences. *Nucleic Acids Res* **52**, D368–D375 (2024).
2. Canner, S. W., Shanker, S. & Gray, J. J. Structure-based neural network protein–carbohydrate interaction predictions at the residue level. *Frontiers in Bioinformatics* **3**, (2023).
3. Bateman, A. *et al.* UniProt: the Universal Protein Knowledgebase in 2023. *Nucleic Acids Res* **51**, D523–D531 (2023).
4. Moore, L. R. *et al.* Revisiting the y-ome of Escherichia coli. *Nucleic Acids Res* **52**, 12201–12207 (2024).
